# HIV-1 group O infection in Cameroon, 1986 to 1998.

**DOI:** 10.3201/eid0703.010321

**Published:** 2001

**Authors:** A Ayouba, P Mauclère, P M Martin, P Cunin, J Mfoupouendoun, B Njinku, S Souquières, F Simon

**Affiliations:** Centre Pasteur du Cameroun, Yaoundé, Cameroon. ayouba@camnet.cm

## Abstract

We report a survey of HIV-1 group O infection in Cameroon during 1986 to 1998. The prevalence of HIV-1/O decreased from 0.6% to 0.4%, while HIV-1/M increased from 19.2% to 31.5% from 1994 to 1998. We concluded that HIV-1/O infection is stable in Cameroon and may be declining slightly.


### VEB-1-Like Extended-Spectrum β-Lactamases in *Pseudomonas aeruginosa*, Kuwait

**Laurent Poirel,* Vincent O. Rotimi,† Eiman M. Mokaddas,† Amal Karim,* and Patrice Nordmann***
*Faculté de Médecine Paris-Sud, Le Kremlin-Bicêtre, France; †Kuwait University, Kuwait

Two clinical *Pseudomonas aeruginosa* isolates from patients in intensive care units in Kuwait were resistant to expanded-spectrum cephalosporins and showed a synergistic effect between ceftazidime and clavulanic acid. This is the first report of extended-spectrum enzymes from nosocomial isolates from the Middle East.

*Pseudomonas aeruginosa* has an inducible, naturally occurring cephalosporinase that confers low-level resistance to aminopenicillins and narrow-spectrum cephalosporins such as cephalothin and cefoxitin (1). Resistance to extended-spectrum cephalosporins may arise from overexpression of this cephalosporinase, acquired beta-lactamases, or both (1). The acquired beta-lactamases may be either clavulanic-acid inhibited (mostly Ambler class A enzymes) or clavulanic-acid resistant (class B and class D enzymes) (2). The class A extended-spectrum beta-lactamases (ESBLs) may derive from narrow-spectrum beta-lactamases of TEM and SHV types, as extensively reported for *Enterobacteriaceae* and rarely for *P. aeruginosa* (2). Other class A enzymes reported in *P. aeruginosa* include PER-1, which we first identified as chromosomally located and which is widespread in *P. aeruginosa* isolates in Turkey (11% of the hospital isolates) (3,4). Lately, another class A ESBL integron-located gene, $bla_{\text{VEB-1}}$, has been identified from *P. aeruginosa* and enterobacterial isolates from Southeast Asia (5-7).

We report on two novel VEB-1-like beta-lactamases from *P. aeruginosa* clinical isolates from Kuwait. This is the first report of extended-spectrum enzymes from nosocomial isolates from this part of the world.

### The Study

*P. aeruginosa* KU-1 was isolated in January 1999 at Ibn Sina Hospital in Kuwait from endotracheal secretions of a 1-day-old infant with respiratory tract infection, hospitalized in the intensive care unit (ICU) unit because of severe enterocolitis. He was first treated with cefotaxime, amikacin, and metronidazole. These antibiotics were discontinued, and he was given imipenem. He improved and was discharged 7 days later. As it was a single isolate of a multidrug-resistant strain, isolation precautions were carried out only in the neonatal ICU. The infant's mother could not remember whether she had received antibiotic therapy during her pregnancy (which was uneventful). She had not traveled outside Kuwait.

*P. aeruginosa* KU-2 was also isolated in June 1999 from the urine of a 73-year-old man admitted to the ICU of another Kuwaiti hospital, Mubarak Al-Kabeer, with ischemic chest pain and bronchiectiasis. On day 2 of his hospitalization, fever developed, but blood and urine cultures were negative. He was treated with ceftazidime for 12 days beginning on day 2. During his hospital stay, hematuria developed, followed by urine retention. On day 28, pus from a urinary catheter infection after transurethral prostatitic surgery grew *P. aeruginosa* KU-2 that was susceptible to norfloxacin. *P. aeruginosa* KU-2 was the only *P. aeruginosa* strain isolated from this patient's clinical specimens. He was treated with norfloxacin. Repeated urine cultures did not yield any organism, and he was discharged 15 days later. He had no history of travel outside Kuwait.

Strains from both patients were identified by using an API-20 NE system (Biomerieux, Marcy-l'Etoile, France). Preliminary antibiotic susceptibility testing by disc diffusion (5) revealed a slight synergy between ceftazidime- and clavulanic acid-containing discs for two clinical isolates, *P. aeruginosa* KU-1 and KU-2. Susceptibility testing of beta-lactams for *P. aeruginosa* KU-1 and KU-2 was then performed by a Mueller-Hinton agar dilution method (8). Both strains showed decreased susceptibility to all beta-lactams except imipenem and piperacillin/tazobactam (Table). MICs of

Table. MICs of beta-lactams for *Pseudomonas aeruginosa* KU-1 and KU-2 clinical isolates, *P. aeruginosa* PU21(pROT-1), and PU21 reference strain

| | MICs (mg/L) | | | |
|---|---|---|---|---|
| Antibiotic[a] | *P. aeruginosa* KU-1 | *P. aeruginosa* KU-2 | *P. aeruginosa* PU21 (pROT-1) | *P. aeruginosa* PU21 |
| Amoxicillin | >512 | >512 | >512 | 64 |
| Ticarcillin | >512 | 512 | 512 | 16 |
| Ticarcillin+CLA | 64 | 16 | 16 | 16 |
| Piperacillin | 64 | 16 | 16 | 2 |
| Piperacillin+TZB | 32 | 8 | 8 | 1 |
| Ceftazidime | 512 | 512 | 512 | 0.25 |
| Ceftazidime+CLA | 16 | 8 | 8 | 0.25 |
| Ceftazidime+TZB | 8 | 4 | 8 | 0.50 |
| Cefotaxime | 512 | 128 | 128 | 4 |
| Cefotaxime+CLA | 64 | 16 | 16 | 2 |
| Imipenem | 2 | 2 | 2 | 0.12 |
| Aztreonam | >512 | >512 | 512 | 0.5 |
| Aztreonam+CLA | 64 | 8 | 8 | 0.5 |

[a]CLA: clavulanic acid at a fixed concentration of 2 mg/L; TZB: tazobactam at a fixed concentration of 4 mg/L.

Address for correspondence: Patrice Nordmann, Service de Bactériologie-Virologie, Hôpital de Bicêtre, 78 rue du Général Leclerc, Le Kremlin-Bicêtre 94275, France; fax: 33 1 45 21 63 40; e-mail: nordmann.patrice@bct.ap-hop-paris.fr ceftazidime were decreased by addition of clavulanic acid and tazobactam, indicating the presence of a clavulanic-acid inhibited ESBL (Table). According to antibiotic susceptibility testing by Mueller-Hinton agar disc diffusion, *P. aeruginosa* KU-1 and KU-2 were also resistant to aminoglycosides (amikacin, gentamicin, netilmicin, tobramycin), chloramphenicol, tetracyclines, sulfonamides, and fluoroquinolones (except for KU-2).

Beta-lactamase extracts from cultures of *P. aeruginosa* KU-1 and KU-2 were obtained (8). Isoelectric focusing analysis (8) revealed beta-lactamases with isoelectric points of 7.4 and 8-8.4, the latter likely corresponding to *P. aeruginosa* AmpC cephalosporinases. Whole-cell DNAs of *P. aeruginosa* KU-1 and KU-2 were then obtained (3). Preliminary polymerase chain reaction (PCR) experiments were performed with DNAs of *P. aeruginosa* KU-1 and KU-2 as templates and primers specific for the following class A beta-lactamases: TEM, SHV, CARB (PSE-1), GES-1, PER-1, and VEB-1 (3,5,9-11). Only PCR using internal primers for $bla_{\text{VEB-1}}$ gave a positive result with an identical 642-bp fragment. External $bla_{\text{VEB-1}}$ specific primers gave 1,070-bp PCR fragments with DNAs of both *P. aeruginosa* strains as templates that were sequenced on both strands (6). The deduced amino acid sequences, obtained over the internet (8), identified VEB-1-like sequences that shared 99% amino acid identity with VEB-1 (Figure). Compared with VEB-1, the amino acid changes in VEB-1a and VEB-1b from *P. aeruginosa* KU-1 and KU-2, respectively, occurred in the putative leader peptide sequence (Figure). Thus, the hydrolytic activity of VEB-1a and VEB-1b should be identical to that reported for VEB-1 beta-lactamase (5).

The genetic background of $bla_{\text{VEB-1a}}$ and $bla_{\text{VEB-1b}}$ was further characterized. Plasmid extraction, conjugation, and electroporation experiments were performed (8). A plasmid (pROT-1) of ca. 70 kb carrying $\text{bla}_{\text{VEB-1a}}$ gene was identified according to hybridization results by using an internal PCR-obtained probe for $bla_{\text{VEB-1}}$ (5). Plasmid pROT-1 was self-conjugative from *P. aeruginosa* KU-1 to in vitro obtained rifampin-resistant *P. aeruginosa* PU21 reference strain after selection of transconjugants onto Mueller-Hinton agar plates each containing 150 µg/mL rifampin and 200 µg/mL ticarcillin. As assessed by antibiotic susceptibility testing by disc diffusion, plasmid pROT-1 conferred additional resistance to gentamicin, netilmicin, sulfonamides, and tobramycin. MICs of beta-lactams for *P. aeruginosa* PU21 (pROT-1) mirrored those obtained for *P. aeruginosa* KU-1 (Table). While the plasmid location of $bla_{\text{VEB-1}}$ gene is known only in Enterobacteriaceae (5-7), its report in *P. aeruginosa* may signal the evolution of its spread. The $bla_{\text{VEB-1b}}$ gene was not plasmid located, but a PCR-obtained 642-bp internal probe for $bla_{\text{VEB-1}}$ hybridized at chromosomal position of whole-cell DNA of *P. aeruginosa* KU-2.

Figure. Comparison of the amino acid sequence of VEB-1, VEB-1a, and VEB-1b beta-lactamases from *Pseudomonas aeruginosa* JES from Thailand and KU-1 and KU-2 from Kuwait. Dashed lines indicate identical amino acids. Arrow indicates position of the putative cleavage site of the peptide leader. Numbering is according to Ambler designation (12).

The $bla_{\text{VEB-1}}$ gene is located on different structures of class 1 integrons (5-7). Integrons comprise two conserved regions (5'-CS and 3'-CS) flanking an internal variable region usually containing several gene cassettes (13). Integrons are in fact expression vectors for antibiotic resistance genes that are included as gene cassettes and are neighbored (13). By using primers located either in the 5'-CS sequence and the 5' end of $bla_{\text{VEB-1}}$ or in the 3' end of $bla_{\text{VEB-1}}$ and the 3'-CS sequence (5,14), PCR amplification experiments were performed with whole-cell DNAs of *P. aeruginosa* KU-1 and KU-2 as templates. In one case (strain KU-1), a PCR fragment was obtained by using $bla_{\text{VEB-1}}$ and 5'-CS primers, indicating that $bla_{\text{VEB-1a}}$ was located downstream of a class 1 integrase gene. In this case, the 4-kb PCR fragment differed from those of known $bla_{\text{VEB-1}}$ containing integrons identified in *Escherichia coli* and *P. aeruginosa* isolates (5-7). Amplimers of 1 kb were obtained for both strains using $bla_{\text{VEB-1}}$ and 3'-CS primers, showing that the 3'-CS end was present in both cases and that the $bla_{\text{VEB-1}}$-like sequences were located next to the 3'-CS end within class 1 integrons. The *attC (59-be)* recombination sites (15) located downstream of gene cassettes were identical for $bla_{\text{VEB-1a}}$ and $bla_{\text{VEB-1b}}$ to those described for $bla_{\text{VEB-1}}$ in *P. aeruginosa* and enterobacterial isolates identified so far from Southeast Asia (5-7). Therefore, an identical $\text{bla}_{\text{VEB-1}}$-like gene cassette may be located on different class 1 integrons. Using 5'-CS and 3'-CS primers, two additional PCR fragments were obtained for each *P. aeruginosa* strain, showing that both strains contained another $bla_{\text{VEB}}$-negative class 1 integron. For *P. aeruginosa* KU-1, a 950-bp PCR fragment for an *aadA1a* gene coding for an aminoglycoside modifying enzyme was found to be plasmid- and integron-located in *Salmonella enterica* serotype Typhimurium (16). For *P. aeruginosa* KU-2, a 500-bp PCR fragment encoding a putative 95 amino acid protein of unknown function was PCR amplified. It shared 71% amino acid identity with an amino acid sequence from a gene that was Tn*1696* transposon-located and In4 integron-located in *P. aeruginosa* (17).

Finally, *P. aeruginosa* KU-1 and KU-2 isolates containing VEB-1-like beta-lactamases were compared with VEB-1 positive *P. aerugionosa* strain JES from Thailand by using random amplified polymorphic DNA technique (10,18). The isolates were not clonally related (data not shown). Although the patients had not traveled outside Kuwait, introduction of *P. aeruginosa* into Kuwaiti hospitals by travelers or patients from Southeast Asia cannot be ruled out.

### Conclusions

The presence of clavulanic-acid inhibited ESBLs in *P. aeruginosa* isolates may account for part of the 50% resistance to ceftazidime of *P. aeruginosa* strains isolates from ICUs in Kuwait (19). ESBLs in *P. aeruginosa* in Kuwait and other Middle Eastern hospitals may be underestimated because routine detection with a double disc synergy test may be difficult. Identification of ESBLs is of interest since they confer resistance to all extended-spectrum cephalosporins and aztreonam, whatever their MICs. This has been confirmed by experimental data using a model of pneumonia in rats with the Ambler class A ESBL, PER-1 (20).

This work underscores that very similar ESBLs may be identified in different parts of the world. It is the first report of ESBL genes characterized from *P. aeruginosa* isolates from the Middle East.

This work was supported by a grant from the Ministères de l'Education Nationale et de la Recherche (UPRES, grant JE-2227), Université Paris XI, Paris, France.

Dr. Poirel is a researcher at Hôpital de Bicêtre, Le Kremlin-Bicêtre, France. He studies the biochemical and genetic mechanisms in beta-lactam resistance.

### References

1. Chen HY, Yuan M, Livermore DM. Mechanisms of resistance to ß-lactam antibiotics amongst *Pseudomonas aeruginosa* isolates collected in the UK in 1993. J Med Microbiol 1995;43:300-9.
2. Nordmann P, Guibert M. Extended-spectrum ß-lactamases in *Pseudomonas aeruginosa*. J Antimicrob Chemother 1998;42:128-31.
3. Nordmann P, Naas T. Sequence analysis of PER-1 extended-spectrum ß-lactamase from *Pseudomonas aeruginosa* and comparison with class A ß-lactamases. Antimicrob Agents Chemother 1994;38:104-14.
4. Vahaboglu H, Ozturk R, Aygun G, Coskunkan F, Yaman A, Kaygusuz A, et al. Widespread detection of PER-type extended-spectrum ß-lactamases among nosocomial *Acinetobacter* and *Pseudomonas aeruginosa* isolates in Turkey: a nationwide multicenter study. Antimicrob Agents Chemother 1997;41:2265-9.
5. Poirel L, Naas T, Guibert M, Chaibi EB, Labia R, Nordmann P. Molecular and biochemical characterization of VEB-1, a novel class A extended-spectrum ß-lactamase encoded by an *Escherichia coli* integron gene. Antimicrob Agents Chemother 1999;43:573-81.
6. Naas T, Poirel L, Karim A, Nordmann P. Molecular characterization of In50, a class 1 integron encoding the gene for the extended-spectrum ß-lactamase VEB-1 in *Pseudomonas aeruginosa*. FEMS Microbiol Lett 1999;176:411-9.
7. Tribuddharat C, Fennewald M. Integron-mediated rifampin resistance in *Pseudomonas aeruginosa*. Antimicrob Agents Chemother 1999;43:960-2.
8. Poirel L, Naas T, Nicolas D, Collet L, Bellais S, Cavallo JD, et al. Characterization of VIM-2, a carbapenem-hydrolyzing metallo-ß-lactamase and its plasmid- and integron-borne gene from a *Pseudomonas aeruginosa* clinical isolate in France. Antimicrob Agents Chemother 2000;44:891-7.
9. Mercier J, Lévesque RC. Cloning of SHV-2, OHIO-1 and OXA-6 ß-lactamase and cloning and sequencing of SHV-1 ß-lactamase. Antimicrob Agents Chemother 1990;34:1577-83.
10. Poirel L, Guibert M, Bellais S, Naas T, Nordmann P. Integron-and carbenicillinase-mediated reduced susceptibility to amoxicillin-clavulanic-acid in isolates of multidrug-resistant *Salmonella enterica* serotype typhimurium DT104 from French patients. Antimicrob Agents Chemother 1999;43:1098-2004.
11. Poirel L, Le Thomas I, Naas T, Karim A, Nordmann P. Biochemical-sequence analyses of GES-1, a novel class A extended-spectrum ß-lactamase, and the class 1 integron In52 from *Klebsiella pneumoniae*. Antimicrob Agents Chemother 2000;44:622-32.
12. Ambler RP. The structure of ß-lactamases. Philos Trans R Soc Lond B Biol Sci 1980;289:321-31.
13. Fluit AC, Schmitz FJ. Class 1 integrons, gene cassettes, mobility, and epidemiology. Eur J Clin Microbiol Infect Dis 1999;18:761-70.
14. Lévesque C, Piché L, Larose C, Roy PH. PCR mapping of integrons reveals several novel combinations of resistance genes. Antimicrob Agents Chemother 1995;39:185-91.
15. Stokes HW, O'Gorman DB, Recchia GD, Parsekhian M, Hall RM. Structure and function of 59-base element recombination sites associated with mobile gene cassettes. Mol Microbiol 1997;26:731-45.
16. Tosini F, Visca P, Luzzi I, Dionisi AM, Pezella C, Petrucca A, et al. Class 1 integron-borne multiple-antibiotic resistance carried by IncFI and IncL/M plasmids in *Salmonella enterica* serotype typhimurium. Antimicrob Agents Chemother 1998;42:3053-8.
17. Hall RM, Brown HJ, Brookes DE, Stokes HW. Integrons found in different locations have identical 5' ends but variable 3' ends. J Bacteriol 1994;176:6286-94.
18. Williams JG, Kubelik AR, Livak KJ, Rafalski JA, Tingey SV. DNA polymorphisms amplified by arbitrary primers are useful as genetic markers. Nucleic Acids Res 1990;18:6531-5.
19. Jamal WY, El Din K, Rotimi VO, Chugh TD. An analysis of hospital-acquired bacteraemia in intensive care unit patients in a university hospital in Kuwait. J Hosp Infect 1999;43:49-56.
20. Mimoz O, Elhelali N, Léotard S, Jacolot A, Laurent F, Samii K, et al. Treatment of experimental pneumonia in rats caused by a PER-1 extended-spectrum ß-lactamase-producing strain of *Pseudomonas aeruginosa*. J Antimicrob Chemother 1999;44:91-7.

## References

[PDF_00275] Ambler R. P. (1980). The structure of beta-lactamases.. Philos Trans R Soc Lond B Biol Sci.

[PDF_00233] Chen H. Y., Yuan M., Livermore D. M. (1995). Mechanisms of resistance to beta-lactam antibiotics amongst Pseudomonas aeruginosa isolates collected in the UK in 1993.. J Med Microbiol.

[PDF_00277] Fluit A. C., Schmitz F. J. (1999). Class 1 integrons, gene cassettes, mobility, and epidemiology.. Eur J Clin Microbiol Infect Dis.

[PDF_00289] Hall R. M., Brown H. J., Brookes D. E., Stokes H. W. (1994). Integrons found in different locations have identical 5' ends but variable 3' ends.. J Bacteriol.

[PDF_00295] Jamal W. Y., El-Din K., Rotimi V. O., Chugh T. D. (1999). An analysis of hospital-acquired bacteraemia in intensive care unit patients in a university hospital in Kuwait.. J Hosp Infect.

[PDF_00279] Lévesque C., Piché L., Larose C., Roy P. H. (1995). PCR mapping of integrons reveals several novel combinations of resistance genes.. Antimicrob Agents Chemother.

[PDF_00263] Mercier J., Levesque R. C. (1990). Cloning of SHV-2, OHIO-1, and OXA-6 beta-lactamases and cloning and sequencing of SHV-1 beta-lactamase.. Antimicrob Agents Chemother.

[PDF_00298] Mimoz O., Elhelali N., Léotard S., Jacolot A., Laurent F., Samii K., Petitjean O., Nordmann P. (1999). Treatment of experimental pneumonia in rats caused by a PER-1 extended-spectrum beta-lactamase-producing strain of Pseudomonas aeruginosa.. J Antimicrob Chemother.

[PDF_00251] Naas T., Poirel L., Karim A., Nordmann P. (1999). Molecular characterization of In50, a class 1 integron encoding the gene for the extended-spectrum beta-lactamase VEB-1 in Pseudomonas aeruginosa.. FEMS Microbiol Lett.

[PDF_00236] Nordmann P., Guibert M. (1998). Extended-spectrum beta-lactamases in Pseudomonas aeruginosa.. J Antimicrob Chemother.

[PDF_00238] Nordmann P., Naas T. (1994). Sequence analysis of PER-1 extended-spectrum beta-lactamase from Pseudomonas aeruginosa and comparison with class A beta-lactamases.. Antimicrob Agents Chemother.

[PDF_00266] Poirel L., Guibert M., Bellais S., Naas T., Nordmann P. (1999). Integron- and carbenicillinase-mediated reduced susceptibility to amoxicillin-clavulanic acid in isolates of multidrug-resistant Salmonella enterica serotype typhimurium DT104 from French patients.. Antimicrob Agents Chemother.

[PDF_00271] Poirel L., Le Thomas I., Naas T., Karim A., Nordmann P. (2000). Biochemical sequence analyses of GES-1, a novel class A extended-spectrum beta-lactamase, and the class 1 integron In52 from Klebsiella pneumoniae.. Antimicrob Agents Chemother.

[PDF_00247] Poirel L., Naas T., Guibert M., Chaibi E. B., Labia R., Nordmann P. (1999). Molecular and biochemical characterization of VEB-1, a novel class A extended-spectrum beta-lactamase encoded by an Escherichia coli integron gene.. Antimicrob Agents Chemother.

[PDF_00258] Poirel L., Naas T., Nicolas D., Collet L., Bellais S., Cavallo J. D., Nordmann P. (2000). Characterization of VIM-2, a carbapenem-hydrolyzing metallo-beta-lactamase and its plasmid- and integron-borne gene from a Pseudomonas aeruginosa clinical isolate in France.. Antimicrob Agents Chemother.

[PDF_00282] Stokes H. W., O'Gorman D. B., Recchia G. D., Parsekhian M., Hall R. M. (1997). Structure and function of 59-base element recombination sites associated with mobile gene cassettes.. Mol Microbiol.

[PDF_00285] Tosini F., Visca P., Luzzi I., Dionisi A. M., Pezzella C., Petrucca A., Carattoli A. (1998). Class 1 integron-borne multiple-antibiotic resistance carried by IncFI and IncL/M plasmids in Salmonella enterica serotype typhimurium.. Antimicrob Agents Chemother.

[PDF_00255] Tribuddharat C., Fennewald M. (1999). Integron-mediated rifampin resistance in Pseudomonas aeruginosa.. Antimicrob Agents Chemother.

[PDF_00242] Vahaboglu H., Oztürk R., Aygün G., Coşkunkan F., Yaman A., Kaygusuz A., Leblebicioglu H., Balik I., Aydin K., Otkun M. (1997). Widespread detection of PER-1-type extended-spectrum beta-lactamases among nosocomial Acinetobacter and Pseudomonas aeruginosa isolates in Turkey: a nationwide multicenter study.. Antimicrob Agents Chemother.

[PDF_00292] Williams J. G., Kubelik A. R., Livak K. J., Rafalski J. A., Tingey S. V. (1990). DNA polymorphisms amplified by arbitrary primers are useful as genetic markers.. Nucleic Acids Res.

